# Long-Term Exposure to Ambient Fine Particulate Matter and Incidence of Major Cardiovascular Diseases: A Prospective Study of 0.5 Million Adults in China

**DOI:** 10.1021/acs.est.2c03084

**Published:** 2022-08-31

**Authors:** Cong Liu, Ka Hung Chan, Jun Lv, Hubert Lam, Katherine Newell, Xia Meng, Yang Liu, Renjie Chen, Christiana Kartsonaki, Neil Wright, Huaidong Du, Ling Yang, Yiping Chen, Yu Guo, Pei Pei, Canqing Yu, Hongbing Shen, Tangchun Wu, Haidong Kan, Zhengming Chen, Liming Li, China Kadorrie Biobank Collaborative Group

**Affiliations:** † School of Public Health, Key Lab of Public Health Safety of the Ministry of Education, NHC Key Lab of Health Technology Assessment, IRDR ICoE on Risk Interconnectivity and Governance on Weather/Climate Extremes Impact and Public Health, Fudan University, Shanghai 200032, China; ‡ Clinical Trial Service Unit & Epidemiological Studies Unit, Nuffield Department of Population Health, University of Oxford, Oxford OX3 7LF, UK; § Oxford British Heart Foundation Center of Research Excellence, University of Oxford, Oxford OX3 7LF, UK; ∥ Department of Epidemiology and Biostatistics, School of Public Health, Peking University, Beijing 100191, China; ⊥ Peking University Center for Public Health and Epidemic Preparedness & Response, Beijing 100191, China; # Key Laboratory of Molecular Cardiovascular Sciences (Peking University), Ministry of Education, Beijing 100191, China; ∇ Gangarosa Department of Environmental Health, Rollins School of Public Health, Emory University, Atlanta, Georgia 30322, United States; ○ Fuwai Hospital Chinese Academy of Medical Sciences, Beijing 100037, China; ◆ Department of Epidemiology, Center for Global Health, School of Public Health, Nanjing Medical University, Nanjing 211166, China; ¶ School of Public Health, Tongji Medical College, Huazhong University of Science and Technology, Wuhan 430030, China; & MRC Population Health Research Unit, Nuffield Department of Population Health, University of Oxford, Oxford OX3 7LF, United Kingdom

**Keywords:** fine particulate matter, cardiovascular disease, incidence, cohort study, satellite-based modeling

## Abstract

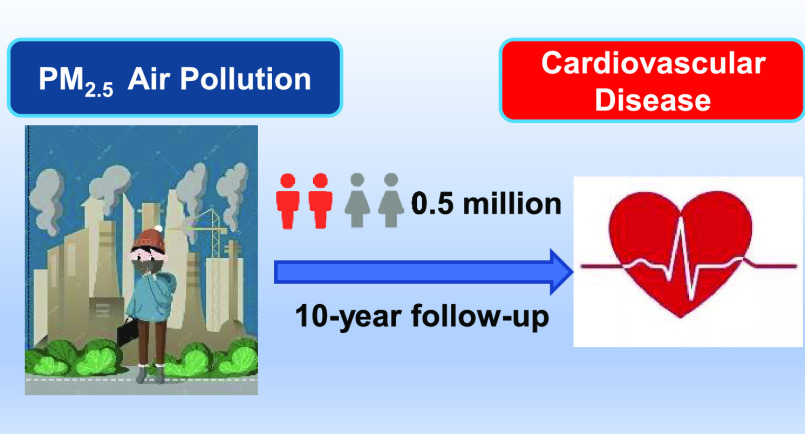

Few cohort studies explored the long-term effects of ambient fine particulate matter (PM_2.5_) on incidence of cardiovascular diseases (CVDs), especially in countries with higher levels of air pollution. We aimed to evaluate the association between long-term exposure to PM_2.5_ and incidence of CVD in China. We performed a prospective cohort study in ten regions that recruited 512,689 adults during 2004–2008, with follow-up until 2017. Annual PM_2.5_ concentrations were estimated using a satellite-based model with national coverage and 1 x 1 km spatial resolution. Time-varying Cox proportional hazard regression models were used to estimate hazard ratios (HRs) for all-cause and cause-specific CVDs associated with PM_2.5_, adjusting for conventional covariates. During 5.08 million person-years of follow-up, 148,030 incident cases of CVD were identified. Long-term exposure to PM_2.5_ showed positive and linear association with incidence of CVD, without a threshold below any concentration. The adjusted HRs per 10 μg/m^3^ increase in PM_2.5_ was 1.04 (95%CI: 1.02, 1.07) for total CVD. The risk estimates differed between certain population subgroups, with greater HRs in men, in household with higher income, and in people using unclean heating fuels. This prospective study of large Chinese population provided essential epidemiological evidence for CVD incident risk associated with PM_2.5_.

## Abstract 2

Limited evidence exists on ambient PM_2.5_ and CVD incidence. This study reports increased risks of CVD incidence associated with long-term exposure to PM_2.5_ in a large prospective cohort in China.

## Introduction

Ambient air pollution poses a significant health risk worldwide.^1^ Among all air pollutants, fine particulate matter (PM_2.5_) with aerodynamic diameters of ≤2.5 μm is considered to be particularly hazardous and has been linked to increased risks of mortality and morbidity from a number of diseases.^2−7^ The newly released Air Quality Guidelines (AQGs) by the World Health Organization (WHO) recommended an annual mean PM_2.5_ concentration of 5 μg/m,^3,^^8^ but over 90% of the world’s population are living in regions that greatly exceed this threshold.^9^ The Global Burden of Disease (GBD) study estimated that ambient PM_2.5_ accounted for over four million deaths in 2019, making it the seventh leading risk factor of global mortality.^10^ Of the global disease burden attributed to ambient PM_2.5_, the majority involves cardiovascular disease (CVD) and populations living in low- and middle-income countries (LMICs) like China.^10^

During the last few decades, numerous epidemiological studies have reported on the associations between PM_2.5_ and CVD, but most tended to focus on its short-term^11−13^ rather than long-term^14,15^ health effects. Moreover, most existing evidence came from North America and Europe, where air pollution levels tend to be much lower compared to LMICs,^16−19^ leaving substantial knowledge gaps in emerging economies at higher ranges of PM_2.5_ exposure, such as China. Furthermore, previous long-term studies have mostly examined mortality outcomes, where the possibility of reverse causality bias cannot be ruled out (i.e., incident diseases may change behavioral risk factors and thus mortality risk during follow-up). In addition, compared to morbidity outcomes, studying mortality alone would suffer from a greater extent of confounding from unaccounted risk factors between incident hospitalization and death.^20^ Few large studies have examined the associations of PM_2.5_ with morbidity outcomes (e.g., incident hospitalizations),^21−23^ which may be triggered over a relatively shorter time period and better reflect the actual impact of ambient PM_2.5_ on disease development.

Despite recent improvements since 2013,^24^ air pollution remains a major public health challenge in China, with PM_2.5_ being the dominant pollutant.^25,26^ Rigorous investigation of the long-term health impact of PM_2.5_ requires reliable longitudinal exposure and health data, both of which had been limited in China until recently. Understanding the relationship between PM_2.5_ exposure and human health is crucial for evidence-based policy making on air quality standards and public health actions. To fill the evidence gap, we presented detailed analyses of PM_2.5_ concentration estimated using exposure assessment models based on satellite remote sensing,^27,28^ with incident risk of CVD in the prospective China Kadoorie Biobank (CKB) of over 0.5 million adults from 10 diverse areas.

## Materials and Methods

### Study Design

The cohort profile of the CKB study has been published elsewhere.^29,30^ The baseline survey was conducted during 2004–2008, and a total of 512,689 adults aged 30–79 years were recruited from 10 geographically defined areas (five urban and five rural regions) of China, including Qingdao (Shandong), Harbin (Heilongjiang), Haikou (Hainan), Suzou (Jiangsu), Liuzhou (Guangxi), Pengzhou (Sichuan), Maiji (Gansu), Huixian (Henan), Tongxiang (Zhejiang), and Liuyang (Hunan). Using a multistage cluster sampling strategy, about 100–150 administrative units (village for rural areas and street committee for urban areas) were randomly selected from local administrative records (*n* = 1.8 million), and all eligible adults (0.5 million) in the selected administrative units were invited to the study (∼30% response rate). In each administrative unit, a survey clinic was established in a central location within 1 km from the residences of most of the eligible participants. In these clinics, trained health workers undertook an electronic questionnaire and physical measurements for all participants following standardized procedures.^29,30^ Detailed information on demographics, lifestyle behavior (such as smoking and drinking), dietary pattern, and medical history was collected. The electronic questionnaire adopted stringent logic and error checks to avoid coding errors or missing data. The data quality was closely monitored during the survey, and health workers were provided with regular feedback and training where appropriate. Approvals were obtained from the Ethical Review Committees of the Chinese Center for Disease Control and Prevention (Beijing, China) and the Oxford Tropical Research Ethics Committee, University of Oxford (Oxford, United Kingdom). All participants provided written informed consent upon recruitment, and the investigation conformed to the principles outlined in the Declaration of Helsinki.

### Assessment of Exposure

We developed a satellite-based exposure assessment model at the national level to predict PM_2.5_ concentrations with spatiotemporal resolutions of 1 km × 1 km, the methodology of which has been published elsewhere.^31^ Briefly, we employed a machine learning modeling approach with random-forest framework. Daily real-time PM_2.5_ records from 2013 to 2017 were obtained from ground monitors, and they were treated as the dependent variable; the multi-angle implementation of atmospheric correction (MAIAC) aerosol optical depth (AOD) retrievals at 1 km × 1 km resolution were used as the main independent variable. We also obtained information on multiple predicting covariates according to previous modeling studies.^32,33^ These variables included the Modern-Era Retrospective Analysis for Research and Applications (version 2) PM_2.5_ prediction predictions, metrological parameters (e.g., temperature, relative humidity, precipitation, and wind speed), land use information (e.g., normalized difference vegetation index), and population density. We integrated two models, one with AOD predictors and another without, when AOD information was missing for the prediction surface. After model training, we compared the PM_2.5_ predictions with out-of-sample ground observations, and the cross-validation results indicated good agreement with an average *R*^2^ of 0.84 and a root mean square error of 16 μg/m^3^. The established model was then utilized to predict PM_2.5_ concentrations over the study period (2005–2017). A map of predicted PM_2.5_ concentration over the study period from 2005–2017 was provided in the Supporting Information (Figure S3). For the exposure assignment, we matched annual mean PM_2.5_ concentrations of each grid cell with the residential geocodes of each participant within their respective clinic location points (each containing 200–300 participants). We also collected data on the temperature of each study region from the China Metrological Administration (http://data.cma.cn/).

### Follow-Up for Morbidity Outcomes

Morbidity outcomes were ascertained through electronic linkage to established morbidity and mortality registries and national health insurance databases (90–99% coverage in the study areas) using unique personal identification numbers of the participants. These databases provided cause-specific fatal and nonfatal events following the 10th revision of the International Classification of Diseases (ICD-10).^29,34^ The endpoints of interest are defined as the first hospitalization event (during the follow-up period) from major CVD, including total CVD (ICD-10; I00–I99), ischemic heart disease (IHD; I20–I25), acute myocardial infarction (MI, I21), total stroke (I60–I61, I63–I64, and I69), hemorrhagic stroke (I61), and ischemic stroke (I63). We also derived composite endpoints of major adverse cardiovascular events (MACE; fatal IHD plus nonfatal MI, IS, or unstable angina; I21–I23, I60–I61, I63, and I64 when nonfatal; I00–I20, I24–I25, I27–I59, I62, I65–I88, and I95–I99 when fatal),^35^ major vascular events (MVE, fatal CVD, I00–I99; nonfatal MI, I21–I23; nonfatal major stroke, I60, I61, I63, I64, I69.0, I69.1, I69.3, and I69.4),^36^ and major coronary events (MCE; fatal IHD plus nonfatal MI; I21–I23; I20, I24, or I25 when fatal) commonly examined in previous cardiovascular epidemiological studies and clinical trials.^36^ Participants without the endpoints of interest were censored upon death, loss to follow-up (*n* = 5302), or 31 December 2017, whichever came sooner.

### Statistical Analysis

The analyses were restricted to incident CVD cases during 2005–2017 as minimal cardiovascular events occurred during the short follow-up period in 2004. We first conducted direct standardization that generated age- and sex-adjusted percentages or means of baseline characteristics by 10 regions. We assessed the associations between long-term exposure to PM_2.5_ and incident cardiovascular events using time-varying Cox proportional hazard regression models, whereby annual concentrations of PM_2.5_ were assigned to each year of follow-up. Compared with conventional approaches that used moving average exposures or fixed exposures (e.g., exposure of the baseline year), this method would reduce the chances of exposure bias during long-term follow-ups.^37^ The hazard ratios (HRs) and 95% confidence intervals (CIs) were estimated for first hospitalization from specific CVD events associated with per 10 μg/m^3^ increase in PM_2.5_ concentrations, adjusting for age, sex, and other potential confounding factors (see below on Models 1–3) and stratified by clinical locations within each of the 10 study areas. The proportional hazard assumption was confirmed by plotting partial residuals against time using standard methods.^38^

We first adjusted for active smoking (never regular-smoker, occasional smoker, ex-regular smoker, or current smoker) and passive smoking (never, former, or present) status in Model 1 as these may be the primary confounders in the PM_2.5_-CVD associations.^39^ Model 2 further adjusted for individual level confounders, including education (no formal school, primary school, middle school, or high school/college/university), body mass index (BMI; two participants with missing BMI values were excluded), self-rated health (excellent, good, fair, or poor), alcohol consumption (never, ex-regular, occasional, monthly, or weekly), total physical activity in the form of metabolic equivalent of tasks (MET) hours (<10, 10–19.9, or >20 h), annual household income (<10,000, 10,000–19,999, 20,000–34,999, or ≥35,000 yuan), solid fuel used for heating (always clean fuels, solid to clean fuels, always solid fuels, never used heating, or others) and cooking (always clean fuels, solid to clean fuels, always solid fuels, never cooked, or others). Model 3 further controlled for annual mean temperature and O_3_, which was referred to as the main model for subsequent analyses. We visualized the exposure–response relationship between PM_2.5_ exposure and cardiovascular incidence from total and specific causes by fitting the concentration of PM_2.5_ with natural spline functions with three degrees of freedom in the main model.^40^

To identify potential effect modifiers, we conducted subgroup analyses by age, sex, educational level (below primary school, middle/high school, above high school), annual household income (<10,000, 10,000–34,999, or ≥35,000 yuan), physical activity (<10, 10–19.9, or ≥20 MET hours), BMI (<18.5, 18.5–24.9, or ≥25), smoking status (never, occasional/ex-regular, or current), alcohol consumption (never, monthly, or weekly), self-rated health (good or poor), cooking and heating fuels (always clean, unclean to clean, or always unclean), and region (urban or rural). Chi-square tests were performed to examine either trend (with 1 df) or heterogeneity (with *n* – 1 df, where *n* = the number of categories) of estimates across subgroups.

In sensitivity analyses, we used alternative exposure sources by substituting PM_2.5_ concentrations for the GBD 2019 exposure estimates with 10 km × 10 km resolution in China.^27^ Moreover, we excluded participants with self-reported history of CVD (i.e., IHD, stroke, or hypertension) at baseline. Furthermore, we excluded participants with poor self-reported health at baseline.^41^

We used R software to perform statistical analyses using the ″survival″ package. The statistical tests were two-sided, and *p*-values of <0.05 were considered statistically significant.

## Results

After standardization by age and sex where appropriate, the distributions of demographic characteristics remained significantly varied across regions with all *p*-values at <0.05 (Table 1). Among the 512,689 participants, the mean (SD) age was 52.0 (10.7) years, 59% were female, 26.4% smoked regularly, and 44.2% were exposed to secondhand smoke. The mean BMI was 23.7 (3.4) kg/m^2^, with 32% being overweight or obese (i.e., BMI > 25 kg/m^2^). Over a third used unclean solid fuels for heating and cooking. Overall, the mean (SD) PM_2.5_ and O_3_ concentrations during the study period (2005–2017) were 52.3 (10.6) μg/m^3^ and 53.9 (6.4) ppb, respectively.

**Table 1:** Characteristics of CKB Participants at Baseline Survey by Study Areas^a^

Variables	Qingdao	Harbin	Haikou	Suzhou	Liuzhou	Pengzhou	Maiji	Huixian	Tongxiang	Liuyang	All
N	35,500	57,548	29,686	53,269	50,174	55,677	49,884	63,353	57,701	59,897	512,689
Age (years)											
30–49	47.7%	39.8%	40.1%	39.7%	32.9%	41.2%	50.6%	45.9%	37.8%	42.0%	41.7%
50–69	45.7%	48.0%	47.6%	53.1%	56.6%	52.5%	44.6%	47.7%	54.8%	50.5%	50.3%
>70	6.6%	12.2%	12.3%	7.2%	10.5%	6.3%	4.9%	6.4%	7.4%	7.5%	8.0%
Mean ± SD	50.8 ± 10.2	53.4 ± 11.4	53.1 ± 11.7	52.1 ± 10.3	54.2 ± 10.4	51.5 ± 10.5	49.4 ± 10.8	50.9 ± 10.4	52.8 ± 9.9	52.1 ± 10.6	52.0 ± 10.7
Sex											
Male	43.6%	39.9%	36.2%	41.9%	38.1%	38.4%	39.9%	44.2%	41.6%	44.1%	41.0%
Female	56.4%	60.1%	63.8%	58.1%	61.9%	61.6%	60.1%	55.8%	58.4%	55.9%	59.0%
BMI (kg/m^2^)											
<18.5	0.8%	2.6%	6.3%	2.6%	3.5%	4.5%	6.4%	2.6%	6.0%	8.4%	4.4%
18.5–24.9	43.6%	55.0%	64.3%	62.0%	63.9%	68.6%	72.2%	58.7%	69.7%	72.3%	63.7%
≥25	55.6%	42.3%	29.4%	35.4%	32.6%	26.8%	21.4%	38.7%	24.3%	19.3%	32.0%
Mean ± SD	25.7 ± 3.5	24.6 ± 3.4	23.3 ± 3.3	24.0 ± 3.2	23.8 ± 3.2	23.3 ± 3.2	22.7 ± 3.1	24.3 ± 3.5	22.9 ± 3.2	22.4 ± 3.1	23.7 ± 3.4
Smoking status											
Non-smoker	65.9%	60.7%	71.9%	61.7%	64.1%	51.5%	63.1%	62.7%	61.6%	62.8%	61.9%
Occasional smoker	2.9%	6.3%	6.1%	4.6%	8.2%	8.1%	4.1%	7.6%	3.8%	4.5%	5.7%
Ex-regular smoker	7.4%	8.4%	4.4%	5.6%	6.3%	7.1%	2.8%	5.7%	7.4%	3.7%	6.0%
Current smoker	23.9%	24.6%	17.6%	28.0%	21.3%	33.3%	29.9%	24.0%	27.2%	29.0%	26.4%
Passive smoking											
Never	38.1%	20.3%	56.6%	14.0%	19.2%	11.4%	18.1%	21.0%	43.4%	21.3%	24.5%
Former	29.2%	45.2%	8.6%	39.7%	48.0%	39.2%	26.3%	30.8%	14.9%	22.3%	31.3%
Present	32.7%	34.5%	34.8%	46.3%	32.8%	49.4%	55.6%	48.2%	41.7%	56.4%	44.2%
Drinking											
Never	37.3%	24.3%	66.0%	59.5%	33.7%	33.4%	65.7%	19.5%	65.0%	63.7%	45.9%
Ex-regular	1.2%	1.0%	1.0%	2.2%	1.3%	3.9%	0.7%	0.5%	2.2%	3.4%	1.8%
Occasional	37.0%	41.5%	23.6%	17.1%	45.2%	31.8%	28.0%	60.6%	13.2%	17.5%	31.8%
Monthly	4.3%	9.7%	2.6%	4.3%	7.3%	6.6%	2.5%	8.7%	3.1%	4.8%	5.7%
Weekly	20.2%	23.5%	6.7%	16.9%	12.5%	24.3%	3.1%	10.7%	16.5%	10.6%	14.9%
Physical activity (MET hours/day)											
<10	8.8%	10.4%	12.5%	4.6%	7.3%	3.6%	3.1%	10.9%	2.5%	4.1%	6.5%
10–19.9	24.9%	22.3%	27.3%	13.2%	21.3%	10.6%	7.4%	21.9%	7.4%	25.3%	17.5%
≥20	66.3%	67.3%	60.2%	82.2%	71.4%	85.8%	89.5%	67.1%	90.1%	70.6%	76.0%
Mean ± SD	18.1 ± 11.4	16.0 ± 10.9	13.6 ± 9.0	25.5 ± 15.2	16.9 ± 11.1	22.1 ± 11.8	28.5 ± 13.0	18.5 ± 15.3	30.2 ± 15.3	17.8 ± 11.4	21.1 ± 13.9
Household income (Yuan)											
<10,000	8.3%	12.5%	21.5%	12.2%	15.1%	62.6%	78.6%	42.1%	6.7%	16.7%	28.2%
10,000-19,999	32.6%	33.3%	31.9%	14.4%	35.7%	28.3%	19.2%	43.7%	14.2%	35.3%	29.1%
20,000-34,999	42.5%	31.5%	22.1%	31.7%	30.3%	5.7%	1.9%	11.5%	41.5%	32.2%	24.7%
≥35,000	16.5%	22.7%	24.6%	41.6%	18.9%	3.4%	0.2%	2.7%	37.6%	15.9%	18.0%
Education											
No formal school	6.4%	3.4%	12.6%	29.6%	3.3%	15.8%	48.6%	14.9%	43.0%	5.6%	18.6%
Primary School	19.2%	9.1%	19.0%	32.1%	18.3%	49.3%	26.3%	36.7%	36.2%	58.6%	32.2%
Middle School	40.7%	31.3%	29.1%	28.4%	34.4%	26.7%	16.5%	34.0%	16.7%	27.0%	28.3%
High School/above	33.7%	56.2%	39.3%	9.9%	44.0%	8.2%	8.6%	14.4%	4.1%	8.8%	21.0%
Self-rated health											
Excellent	21.7%	30.6%	11.2%	29.0%	14.8%	6.9%	21.2%	12.4%	15.6%	12.7%	17.6%
Good	29.3%	19.1%	20.7%	32.0%	18.4%	30.6%	28.0%	31.1%	43.6%	24.1%	28.1%
Fair	43.8%	40.0%	61.4%	28.6%	56.5%	41.5%	39.2%	42.9%	36.2%	56.0%	43.9%
Poor	5.3%	10.3%	6.6%	10.4%	10.3%	20.9%	11.6%	13.6%	4.5%	7.2%	10.4%
Heating fuels											
Always clean	4.1%	28.2%	0.1%	18.9%	13.9%	16.4%	0.1%	0.2%	0.6%	0.3%	8.6%
Unclean to clean	32.0%	62.4%	0.0%	0.7%	7.5%	2.6%	0.8%	1.1%	0.0%	5.8%	11.3%
Always unclean	59.7%	4.3%	0.1%	0.1%	8.7%	6.5%	94.2%	81.9%	0.0%	92.9%	36.2%
Never used heating	0.2%	0.0%	97.8%	79.1%	58.1%	70.3%	0.4%	2.6%	99.3%	0.5%	38.9%
Others	3.9%	5.1%	2.1%	1.3%	11.8%	4.2%	4.5%	14.2%	0.1%	0.6%	5.0%
Cooking fuels											
Always clean	56.4%	38.7%	31.7%	15.2%	34.1%	4.6%	0.4%	0.6%	14.0%	0.5%	16.9%
Unclean to clean	22.0%	33.9%	17.8%	45.7%	42.9%	9.3%	1.0%	0.8%	14.9%	1.6%	19.0%
Always unclean	0.4%	1.3%	14.1%	14.5%	3.0%	55.5%	58.6%	63.0%	35.2%	65.4%	34.2%
Never cooked	17.2%	20.8%	31.9%	15.5%	11.8%	26.6%	37.8%	30.4%	33.7%	29.3%	25.1%
Others	3.9%	5.4%	4.5%	9.0%	8.2%	4.0%	2.1%	5.2%	2.2%	3.1%	4.8%
PM_2.5_ (μg/m^3^)											
Mean ± SD	57.1 ± 0.9	55.3 ± 1.3	26.3 ± 0.4	57.7 ± 1.6	45.3 ± 0.9	51.4 ± 4.0	39.1 ± 2.0	70.9 ± 1.4	53.6 ± 1.1	51.3 ± 1.7	52.3 ± 10.6
O_3_ (ppb)											
Mean ± SD	52.3 ± 2.4	43.3 ± 0.3	43.4 ± 1.3	59.6 ± 0.7	47.3 ± 0.7	59.7 ± 1.8	53.8 ± 0.4	60.6 ± 1.4	59.7 ± 1.0	53.0 ± 1.4	53.9 ± 6.4

a Abbreviations: BMI, Body mass index; MET-hours, metabolic equivalent task hours. Note: All variables were adjusted by the age and sex of the study population where appropriate. Two-sided P values were derived from ANOVA for continuous variables and from the Chi-square test for categorical variables, all P values were < 0.005.

During 5.08 million person-years of follow-up (mean of 9.9 [SD = 3.4] years), 148,030 were hospitalized for CVD (Table S1), including 50,323 from IHD, 4604 from AMI, and 57,222 from stroke (50,174 from ischemic stroke and 7684 from hemorrhagic stroke). Among the 10 study areas, the annual number of CVD cases increased gradually (Table S2). Harbin had the highest number of CVD events (27,859).

**Figure 1:**
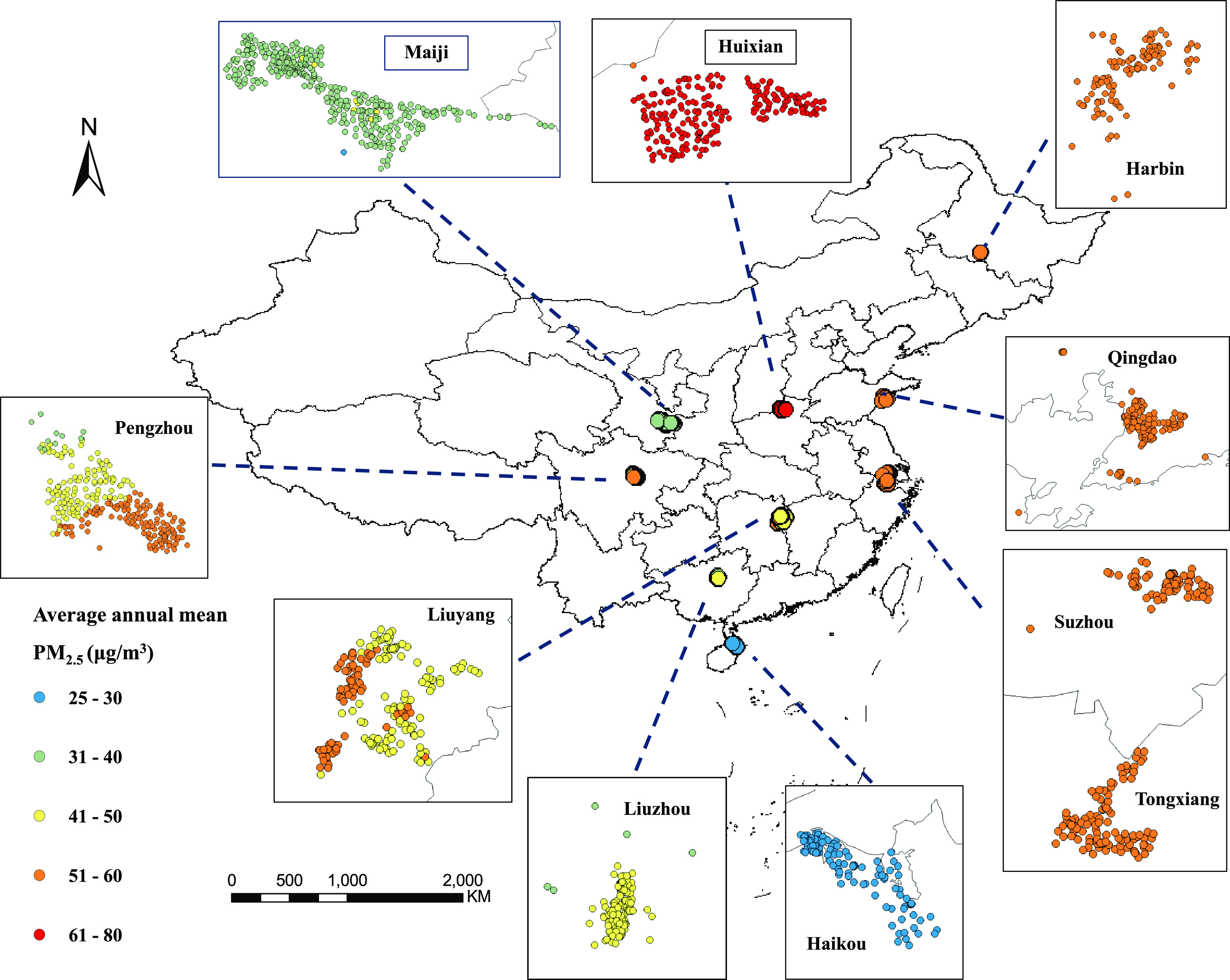
Geographical locations of residence of study participants in 10 areas of CKB cohort and estimated average annual PM_2.5_ concertation.

Figure 1 illustrates the clinical locations of the of study participants in 10 study areas and the respective average annual mean exposure to PM_2.5_. Across the 10 study areas, there were more than three-fold variations in PM_2.5_, from 24.9 in Haikou to 78.8 μg/m^3^ in Huixian (Figure 1). Within the specific study area (Figure S1), however, the exposure variations were small: the urban region Haikou had the lowest PM_2.5_ level with a mean of 26.1 μg/m^3^ (24.9–26.7 μg/m^3^), and we observed the highest level of PM_2.5_ in the rural region Huixian with a mean of 70.8 μg/m^3^ (61.9–78.8 μg/m^3^).

**Figure 2:**
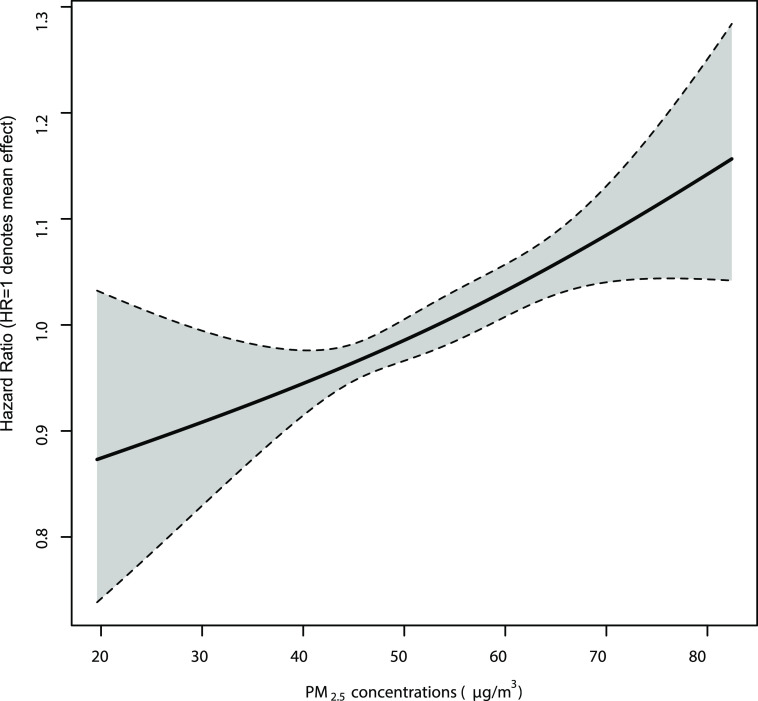
Concentration-response curves for long-term exposure to PM_2.5_ and risk of cardiovascular incidence. The vertical scale can be interpreted as the relative ratio of the mean effect of PM_2.5_ on CVD, and the fraction of the curve below HR = 1 denotes a smaller estimate compared with the mean effect. Covariates were adjusted as main models, controlling for age, sex, active/passive smoking status, education, BMI, self-rated health, alcohol consumption, physical activity, household income, cooking/heating fuels, ozone and temperature, except for strata indicators.

**Figure 3:**
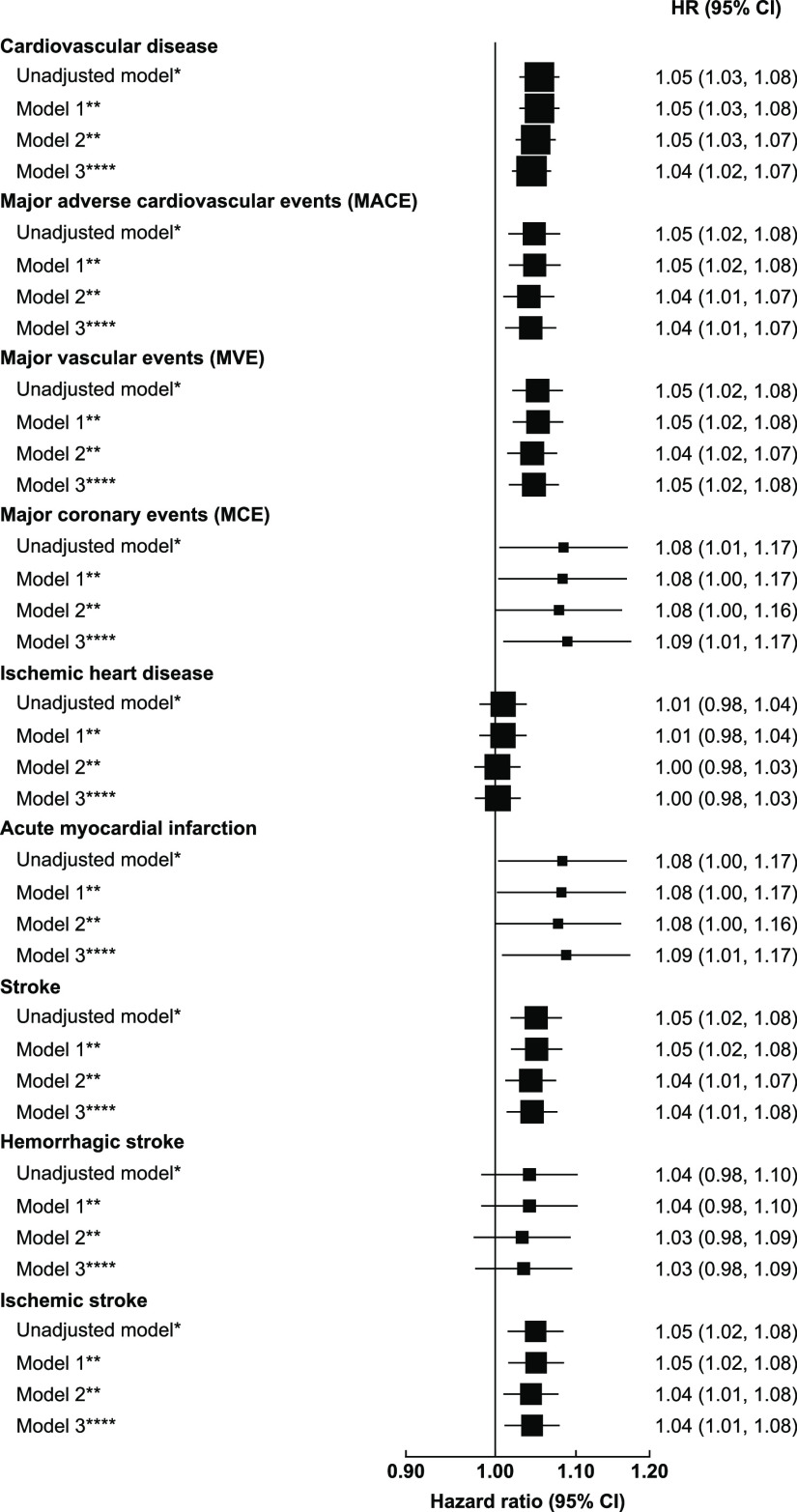
Adjusted hazard ratios of major cardiovascular diseases associated with per 10 μg/m^3^ increase in PM_2.5_ concentrations. The black boxes represent hazard ratios (HRs), with the size inversely proportional to the variance of the logarithm of the HRs, and the horizontal lines represent 95% confidence intervals(CI). The arrows represent a negative HR < 1 and its 95% CI. Notes for models: Unadjusted model*Adjusting for age and sex only. Model 1**Adjusting for age, sex, active smoking status, passive smoking status. Model 2*** Adjusting for age, sex, active/passive smoking status, education, BMI, self-rated health, alcohol consumption, physical activity, household income, solid fuel used for cooking/heating. Model 3**** Adjusting for age, sex, active/passive smoking status, education, BMI, self-rated health, alcohol consumption, physical activity, household income, solid fuel used for cooking/heating, ozone and temperature.

Long-term exposure to PM_2.5_ showed significantly positive and apparently linear associations with increased risks of CVD (Figure 2). The concentration–response curve appeared almost linear and increasing with no obvious threshold across the 20–85 μg/m^3^ range. The same pattern was observed for AMI, hemorrhagic stroke, and MCE (Figure S2), whereas for other specific cardiovascular diseases, the slopes at lower ranges of exposure could be flat (i.e., IHD, stroke, and MACE), but the confidence intervals were very wide. The effect estimates were generally robust to different levels of adjustment (Figure 3). In the main models (Model 3, for a 10 μg/m^3^ increase in PM_2.5_ concentrations), the adjusted HRs (95%CI) were 1.04 (1.02, 1.07), 1.09 (1.01, 1.17), 1.04 (1.01, 1.08), and 1.04 (1.01, 1.08) for total CVD, AMI, stroke, and ischemic stroke, respectively. For IHD and hemorrhagic stroke, there were also positive but non-significant associations. Furthermore, we observed similar results for MACE, MVE and MCE, with HRs of 1.04 (1.01, 1.07), 1.05 (1.02, 1.08), and 1.04 (1.01, 1.08), respectively. The region-specific associations between PM_2.5_ and CVD are presented in Table S3. We observed positive and significant associations between long-term PM_2.5_ exposure and CVD in Qingdao, Harbin, Maiji, Tongxiang, and Huixian, whereas the estimates in Liuzhou, Suzhou, Haikou, Pengzhou, and Liuyang were nonsignificant.

**Figure 4:**
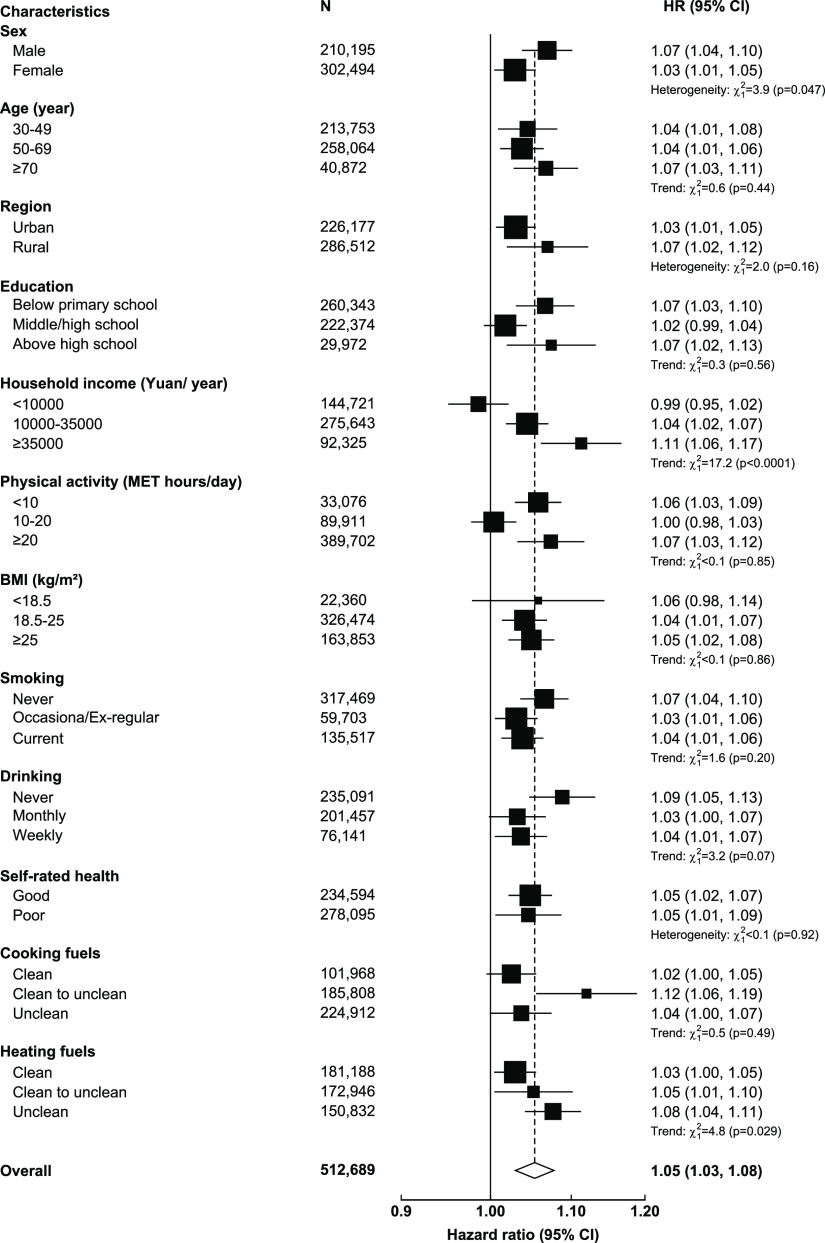
Adjusted hazard ratios of cardiovascular incidence associated with per 10 μg/m^3^ in PM_2.5_ concentrations in selected population subgroups. The black boxes represent hazard ratios (HRs), with the size inversely proportional to the variance of the logarithm of the HRs, and the horizontal lines represent 95% confidence intervals(CI). The open diamond represents the overall HR and 95% CI. Chi-square tests were performed to examine either trend (with 1 df) or heterogeneity (with n-1 df, where n = the number of categories) of HR per 10 μg/m^3^ PM_2.5_ across subgroups.

In stratified analyses (Figure 4), we observed several potential effect modifiers in the PM_2.5_-CVD associations. For example, the risk estimates for males, households with higher income, or those using unclean fuels for heating were significantly larger than their counterparts (*p*-values for trend < 0.05). Similarly, though with non-significant between-group differences, we observed different PM_2.5_-CVD associations by age, alcohol drinking, and region. There was no sign of effect modification by educational levels, physical activity, BMI, and self-rated health.

In sensitivity analyses (Table 2), the exchanged GBD PM_2.5_ predictions with lower spatial resolutions yielded positive but weaker associations with CVDs than our exposure products. In the analyses that excluded participants with self-reported baseline prevalence of cardiovascular-related diseases or those with poor self-reported health, the main results were not altered materially.

**Table 2:** Sensitivity Analyses on Hazard Ratios (HRs and 95%CI) of Cardiovascular Incidence Associated with per 10 μg/m^3^ in PM_2.5_ Concentrations^a^

Models	Main model	Sensitivity analysis 1	Sensitivity analysis 2	Sensitivity analysis 3
Cardiovascular disease	1.04 (1.02, 1.07)	1.00 (1.00, 1.01)	1.04 (1.02, 1.07)	1.05 (1.03, 1.07)
MACE	1.04 (1.01, 1.07)	1.00 (1.00, 1.00)	1.04 (1.01, 1.07)	1.04 (1.01, 1.08)
MVE	1.05 (1.02, 1.08)	1.00 (1.00, 1.01)	1.05 (1.02, 1.08)	1.05 (1.02, 1.08)
MCE	1.09 (1.01, 1.17)	1.00 (0.99, 1.01)	1.09 (1.01, 1.17)	1.06 (0.97, 1.15)
IHD	1.00 (0.98, 1.03)	1.00 (1.00, 1.00)	1.00 (0.98, 1.03)	1.01 (0.98, 1.04)
AMI	1.09 (1.01, 1.17)	1.00 (0.99, 1.01)	1.09 (1.01, 1.17)	1.06 (0.97, 1.15)
Stroke	1.04 (1.01, 1.08)	1.00 (1.00, 1.01)	1.04 (1.01, 1.08)	1.05 (1.01, 1.08)
Hemorrhagic stroke	1.03 (0.98, 1.09)	1.00 (1.00, 1.01)	1.03 (0.98, 1.09)	1.03 (0.97, 1.10)
Ischemic stroke	1.04 (1.01, 1.08)	1.00 (1.00, 1.01)	1.04 (1.01, 1.08)	1.05 (1.01, 1.08)

a Abbreviations: MACE, major adverse cardiovascular events; MVE, major vascular events; MCE, major coronary events; IHD, ischemic heart disease; AMI, acute myocardial infraction. Notes: Main model was adjusted for age, sex, active/passive smoking status, education, BMI, self-rated health, alcohol consumption, physical activity, household income, solid fuel used for cooking/heating, ozone and temperature. Sensitivity analysis 1, using substitute PM_2.5_ concentrations from GBD 2019 exposure estimates. Sensitivity analysis 2, excluding self-reported baseline prevalence of coronary heart disease, stroke and hypertension. Sensitivity analysis 3, excluding participants with poor self-reported health at baseline.

## Discussion

This large prospective cohort study demonstrated significantly increased risk of incident CVD associated with long-term exposure to ambient PM_2.5_ in China. Specific causes of CVD, including AMI, stroke (ischemic stroke in particular), MACE, MVE, and MCE, were also linked with PM_2.5_ exposure with similar effect estimates. The concentration–response curve was positive and broadly linear for the PM_2.5_-CVD association. We also identified potential effect modifiers by sex, household income, and fuel used for heating.

To the best of our knowledge, this was one of the few studies on the long-term health effects of ambient PM_2.5_ on cardiovascular incidence, and our estimates for total CVD (HR = 1.04, 1.02–1.07) associated with a 10 μg/m^3^ increase in PM_2.5_ concentrations appeared much more modest compared with those reported in other studies. Miller et al. examined the long-term association between PM_2.5_ and cardiovascular incidence in 65,893 postmenopausal women (mean age of 63 years, SD = 7.3) in the Women’s Health Initiative Observational Study^42^ and found an HR of 1.24 (1.09, 1.41) per 10 μg/m^3^ increase in PM_2.5_. Liang et al. used follow-up data from the Prediction for Atherosclerotic Cardiovascular Disease Risk in China (China-PAR) study and reported a similar HR of 1.25 (95% CI: 1.22, 1.28) for CVD incidence. Notably, both cohorts were specifically designed either for older women or hospital cardiovascular inpatients who were initially at higher risk of CVD, while our study was based on a general population aged 30 years or above. As PM_2.5_ has been shown to be more harmful to high-risk individuals or the elderly,^43−45^ the above demographic and risk profile differences may explain the weaker association observed in our study. Furthermore, the relative smaller effect estimates in the current analysis might be supported by the exposure–response relationships in two well-established models. First, the Integrated Exposure–Response (IER) model developed for the GBD study, which integrated four types of PM_2.5_ exposures (outdoor PM_2.5_, active smoking, secondhand smoking, and household burning of solid fuels) associated with six specific causes of death (ischemic heart disease, stroke, chronic obstructive pulmonary disease, lung cancer, lower respiratory infection, and type 2 diabetes).^46^ Second, the Global Exposure Mortality Model (GEMM), which modeled the shape of the association between PM_2.5_ and nonaccidental mortality using data from 41 cohorts from 16 countries.^47^ Both models have exhibited smaller exposure–response relationships at a higher range of concentrations compared with lower ones, indicating a potential smaller effect of PM_2.5_ in regions with high exposures. As a study conducted in China with very high air pollution levels, the effect estimates for CVD per unit in PM_2.5_ exposure may be lower. In addition, this analysis covered a prolonged time period from 2005 to 2017. Since the air pollution levels dropped dramatically in China after 2013, the reverse trend of increasing CVD cases and declining PM_2.5_ concentrations may also affect the effect estimates.

As CVD is a top cause of mortality,^48^ we compared the magnitude of effect with previous studies on long-term effects of PM_2.5_ on total mortality, and our estimates were generally a bit smaller. For example, Di et al. investigated the US Medicare population that lives in very low levels of air pollution and found a HR of 1.073 (95% CI: 1.071, 1.075) for total mortality associated with a 10 μg/m^3^ increment in PM_2.5_. The European Study of Cohorts for Air Pollution Effects (ESCAPE) employed land use regression models in 22 countries and estimated increased risks of natural-cause mortality with an HR of 1.14 (95% CI:1.04, 1.26). Two recent cohort studies in China, the Chinese Longitudinal Healthy Longevity Survey (CLHLS)^49^ and the Chinese Men Study,^50^ respectively reported HRs of 1.08 (95% CI: 1.06, 1.09) and 1.09 (95%CI: 1.08, 1.09) associated with a 10 μg/m^3^ increase in PM_2.5_. Apart from these, the extended analyses of the classical air pollution cohort studies in the US also found similar results for PM_2.5_ and CVD mortality, such as the Harvard Six-City Study (HR = 1.26, 95%CI: 1.14, 1.40)^51^ and the America Cancer Society cohort (HR = 1.12, 95%CI: 1.10, 1.15).^52^ Nonetheless, the effect estimates from studies with different types of endpoints may not be comparable, especially when other uncertainties exist due to differences in PM_2.5_ composition, population characteristics, and exposure patterns.^6,53^ Nevertheless, our findings reinforced the significant health effects of PM_2.5_ on the cardiovascular system.

For specific CVDs, we observed significant associations of PM_2.5_ exposure with total stroke and ischemic stroke but not hemorrhagic stroke, which is broadly consistent with previous studies.^54^ These phenomena can be explained by the well-reported biological mechanism that PM_2.5_ exposure induces oxidative stress and systematic inflammation, both of which are involved in myocardial ischemia.^55^ In contrast, the etiology of hemorrhagic stroke is less well-understood (except for blood pressure), and it has been suggested that PM_2.5_ exposure may not play an important role in predisposing such acute and progressive disease.^54^ Furthermore, ischemic stroke accounts for a larger proportion in total stroke rather than hemorrhagic ones, and the larger study sample may contribute to larger statistical power for a significant finding. In addition, some of the effect estimates were quite similar, which could be explained by the fact that some of the endpoints are overlapped or combined from specific causes (i.e., MACE, MCE, and MVE).

Our stratification analyses observed certain trends across subgroups that were generally consistent with previous reports, which may provide additional insights for the identification of susceptible factors. First, the effect estimate appeared slightly larger in males, which is in line with most investigations that found higher risks of cardiovascular diseases in male population.^14,56^ We also observed slightly larger associations in the older age groups. However, the IER and GEMM both observed smaller slopes of associations in populations with higher age.^46,47^ It has also been reported that older age groups experience greater absolute risk of mortality associated with PM_2.5_ but lower relative risk.^6^ More studies are needed for a resolved conclusion. Then, the HRs for CVD became larger with higher household income, and this may be explained by the usual pattern that wealthier people are from more developed areas and may thus be exposed to higher levels of exposure. Interestingly, we found somewhat stronger associations of PM_2.5_ with CVD in non-smokers and non-drinkers. The same observation has been reported by Liang et al. and a previous study in the US.^14,57^ A plausible hypothesis is that smoking and drinking may share similar exposure pathways and toxicities with inhalation of PM_2.5_ such as oxidative stress and inflammation.^57^ In these circumstances, smoking and drinking behavior may have dominated the main contribution to CVD development; thus, additional exposure to PM_2.5_ may show a smaller effect.^58,59^ Last, there were larger PM_2.5_-CVD associations in rural areas, and participants that used unclean cooking and heating fuels, which on one hand verified the previous findings on the adverse effect of solid fuel use on human health^41,60^ and on the other hand indicated potential synergistic effect of indoor air pollution with ambient PM_2.5_ exposure.

Our sensitivity analyses demonstrated no material changes after excluding participants with self-reported prevalent CVD or poor self-rated health. Notably, when we alternatively used another source of PM_2.5_ predictions from the GBD database, the associations of PM_2.5_ with CVD remained positive, but the effect estimates were somewhat smaller. We postulate that the relatively lower spatial resolution (10 km × 10 km) compared with our primary models (1 km × 1 km) might have further reduced the exposure variations between participants within each region, subsequently leading to smaller central estimates for the PM_2.5_-CVD associations.

Our study offers substantial policy implications. First, we successfully utilized a national-scale exposure assessment model to predict historical exposure levels for established long-term cohorts, and this strategy can be generalized and adopted by future epidemiological investigations. In addition, robust associations of PM_2.5_ air pollution with several major causes of CVD were identified, such as AMI, ischemic stroke, and MACE, which arouses attention on these environmental-related risk factors for patients. Furthermore, there was a larger risk of CVD in older people, rural areas, and those exposed to unclean cooking/heating fuels; thus, it is warranted to implement intervention programs for these susceptible factors/populations. Last, as illustrated in the exposure–response curve, the PM_2.5_-CVD association was present across the concentration range without a clear threshold and even below the well below the current recommended levels of PM_2.5_ concentration (35 μg/m^3^) in China.^61^ This reaffirms the importance of further revising the current air quality standards in China to continuously improve air quality for greater public health benefits.

The major strengths of this study lie in the large number of study participants over 0.5 million, providing enough statistical power to produce robust estimates and ensuring the generalizability of the findings. Furthermore, the high diagnostic quality of incidence endpoints in the CKB cohort benefited the investigation on the first hospitalization event for a wide range of CVDs. For example, over 95% stroke cases were confirmed by brain imaging, and we have also undertaken independent outcome adjudication via retrieval and review of original medical records. We also observed high consistency of concentration–response relationships across these diseases and among different population subgroups. Our study adds to the scarce scientific knowledge on long-term exposure to PM_2.5_ and CVD incidence in developing regions with high exposure levels. Moreover, we self-developed a high-resolution exposure assessment model with long coverage time, which facilities the prediction of historical PM_2.5_ exposures in subsequent studies.

However, some limitations exist for the present study that need consideration. First, as CVD has a prolonged development period, it may be difficult to precisely determine its temporality. Yet, the primary aim of this analysis was to establish the link between PM_2.5_ exposure and CVD incidence rather than the exact timing of the development of incident cases. Second, although a high-resolution model was used, exposure misclassification was inevitable as exposure assignment was realized on a cluster level, and we could not characterize time activity patterns or the existence of residential mobility for each individual. Further, given the small number of regions covered and clustered within the particular study region, there is a general lack of exposure variation, and our observation could be due partly to ecological fallacy. Third, due to lack of data, we could only assess the confounding effect of O_3_ but not for other co-pollutants (i.e., nitrogen dioxide), and this could be enhanced in future studies with increased data availability. Fourth, as in most previous studies, residual confounding, especially from socioeconomic status, may remain despite the adjustment made. Fifth, as in most prospective cohort studies, participation was voluntary and we were not able to collect information on non-respondents; thus, healthy volunteer bias was inevitable. However, as discussed by Manolio et al. and Rothman et al.,^62,63^ epidemiological studies assessing etiological questions with substantial sample sizes and heterogeneity in exposure should generate evidence that are reasonably applicable to similar populations.

In conclusion, this large prospective cohort study in China identified significantly increased risks of total and cause-specific CVD incidence with long-term exposure to PM_2.5_, which reinforced the previous evidence on PM_2.5_-CVD association. The monotonically increasing and no-threshold concentration–response relationship suggests a motivation to further tighten the recommended level of PM_2.5_. Our findings may provide essential epidemiological evidence for developing countries with higher levels of air pollution and may have certain policy implications for continuously improving air quality for better public health welfare and achieving sustainable development in China.

## Supporting Information Available

Summaries of incident CVD cases, region-specific estimates for PM_2.5_-CVD associations, boxplots of region-specific exposures, concentration–response curves for specific CVDs, and prediction map of PM_2.5_ (PDF)

## Notes

^◊^ The members of the China Kadorrie Biobank Collaborative Group are listed in the Supporting Information.

## Abbreviations

BMI: body mass index
MET-hours: metabolic equivalent task hours
*N*: number of participants in each stratum (covariates were adjusted as main models, controlling for age, sex, active/passive smoking status, education, BMI, self-rated health, alcohol consumption, physical activity, household income, cooking/heating fuels, ozone, and temperature, except for strata indicators)
