# Supplementary material for: Long-Term Exposure to Ambient Fine Particulate Matter and Incidence of Major Cardiovascular Diseases: A Prospective Study of 0.5 Million Adults in China
Source: Environ Sci Technol. 2022 Aug 31;56(18):13200–11. doi: 10.1021/acs.est.2c03084 (PMC9494741; doi:10.1021/acs.est.2c03084)
Supplement: Supplementary file 1 — es2c03084_si_001.pdf [file es2c03084_si_001.pdf]

Supporting Information

Long-term exposure to ambient fine particulate matter and incidence of major cardiovascular diseases: a prospective study of 0.5 million adults in China

Cong Liu[1,†], Ka Hung Chan[2,3,†], Jun Lv[4,5,6,†], Hubert Lam[2], Katherine Newell[2], Xia Meng[1], Yang Liu[7], Renjie Chen[1], Christiana Kartsonaki[2], Neil Wright[2], Huaidong Du[2], Ling Yang[2], Yiping Chen[2], Yu Guo[8], Pei Pei[8], Canqing Yu[4,5], Hongbing Shen[9], Tangchun Wu[10], Haidong Kan[1,*], Zhengming Chen[2,11,*], Liming Li [4,5*], China Kadorrie Biobank Collaborative Group¶

Affiliations:

[1] School of Public Health, Key Lab of Public Health Safety of the Ministry of Education, NHC Key Lab of Health Technology Assessment, IRDR ICoE on Risk Interconnectivity and Governance on Weather/Climate Extremes Impact and Public Health, Fudan University, Shanghai, 200032, China;

[2] Clinical Trial Service Unit & Epidemiological Studies Unit, Nuffield Department of Population Health, University of Oxford, Oxford, OX3 7LF, UK;

[3] Oxford British Heart Foundation Center of Research Excellence, University of Oxford, Oxford, OX3 7LF UK

[4] Department of Epidemiology and Biostatistics, School of Public Health, Peking University, Beijing, 100191, China;

[5] Peking University Center for Public Health and Epidemic Preparedness & Response, Beijing, 100191, China;

[6] Key Laboratory of Molecular Cardiovascular Sciences (Peking University), Ministry of Education, Beijing, 100191, China;

[7] Gangarosa Department of Environmental Health, Rollins School of Public Health, Emory University, Atlanta, GA 30322, US;

[8] Fuwai Hospital Chinese Academy of Medical Sciences, Beijing, 100037, China;

[9] Department of Epidemiology, Center for Global Health, School of Public Health,

Nanjing Medical University, Nanjing, 211166, China;

[10] School of Public Health, Tongji Medical College, Huazhong University of Science and Technology, Wuhan, 430030, China;

[11] MRC Population Health Research Unit, Nuffield Department of Population Health, University of Oxford, OX3 7LF, UK.

† Cong Liu, Ka Hung Chan, and Jun Lv contributed equally to this work.
¶ Members of China Kadorrie Biobank Collaborative Group in Supplemental Material.

Address for correspondence: Prof. Liming Li, Department of Epidemiology and Biostatistics, School of Public Health, Peking University, Beijing, China. Email: lmlee@bjmu.edu.cn; Prof. Zhengming Chen, Clinical Trial Service Unit & Epidemiological Studies Unit, Nuffield Department of Population Health, University of Oxford, Oxford, UK. Email: zhengming.chen@ndph.ox.ac.uk; Prof. Haidong Kan, Department of Environmental Health, School of Public Health, Fudan University, No.130 Dong-An Road, Shanghai, 200032, China. Email: kanh@fudan.edu.cn.

¶ **Members of the China Kadoorie Biobank collaborative group:**

International Steering Committee: Junshi Chen, Zhengming Chen (PI), Robert Clarke, Rory Collins, Yu Guo, Liming Li (PI), Jun Lv, Richard Peto, Robin Walters. **International Co-ordinating Center, Oxford:** Daniel Avery, Ruth Boxall, Derrick Bennett, Yumei Chang, Yiping Chen, Zhengming Chen, Robert Clarke, Huaidong Du, Simon Gilbert, Alex Hacker, Mike Hill, Michael Holmes, Andri Iona, Christiana Kartsonaki, Rene Kerosi, Ling Kong, Om Kurmi, Garry Lancaster, Sarah Lewington, Kuang Lin, John McDonnell, Iona Millwood, Qunhua Nie, Jayakrishnan Radhakrishnan, Paul Ryder, Sam Sansome, Dan Schmidt, Paul Sherliker, Rajani Sohoni, Becky Stevens, Iain Turnbull, Robin Walters, Jenny Wang, Lin Wang, Neil Wright, Ling Yang, Xiaoming Yang. **National Co-ordinating Center, Beijing:** Yu Guo, Xiao Han, Can Hou, Jun Lv, Pei Pei, Chao Liu, Canqing Yu. **10 Regional Co-ordinating Centers: Qingdao CDC:** Zengchang Pang, Ruqin Gao, Shanpeng Li, Shaojie Wang, Yongmei Liu, Ranran Du, Yajing Zang, Liang Cheng, Xiaocao Tian, Hua Zhang, Yaoming Zhai, Feng Ning, Xiaohui Sun, Feifei Li. **Licang CDC:** Silu Lv,

Junzheng Wang, Wei Hou. **Heilongjiang Provincial CDC:** Mingyuan Zeng, Ge Jiang, Xue Zhou. **Nangang CDC:** Liqiu Yang, Hui He, Bo Yu, Yanjie Li, Qinai Xu,Quan Kang, Ziyan Guo. **Hainan Provincial CDC:** Dan Wang, Ximin Hu, Jinyan Chen, Yan Fu, Zhenwang Fu, Xiaohuan Wang. **Meilan CDC:** Min Weng, Zhendong Guo, Shukuan Wu,Yilei Li, Huimei Li, Zhifang Fu. **Jiangsu Provincial CDC:** Ming Wu, Yonglin Zhou, Jinyi Zhou, Ran Tao, Jie Yang, Jian Su. **Suzhou CDC:** Fang liu, Jun Zhang, Yihe Hu, Yan Lu, Liangcai Ma, Aiyu Tang, Shuo Zhang, Jianrong Jin, Jingchao Liu. **Guangxi Provincial CDC:** Zhenzhu Tang, Naying Chen, Ying Huang. **Liuzhou CDC:** Mingqiang Li, Jinhuai Meng, Rong Pan, Qilian Jiang, Jian Lan,Yun Liu, Liuping Wei, Liyuan Zhou, Ningyu Chen Ping Wang, Fanwen Meng, Yulu Qin,, Sisi Wang. **Sichuan Provincial CDC:** Xianping Wu, Ningmei Zhang, Xiaofang Chen,Weiwei Zhou. **Pengzhou CDC:** Guojin Luo, Jianguo Li, Xiaofang Chen, Xunfu Zhong, Jiaqiu Liu, Qiang Sun. **Gansu Provincial CDC:** Pengfei Ge, Xiaolan Ren, Caixia Dong. **Maiji CDC:** Hui Zhang, Enke Mao, Xiaoping Wang, Tao Wang, Xi zhang. **Henan Provincial CDC:** Ding Zhang, Gang Zhou, Shixian Feng, Liang Chang, Lei Fan. **Huixian CDC:** Yulian Gao, Tianyou He, Huarong Sun, Pan He, Chen Hu, Xukui Zhang, Huifang Wu, Pan He. **Zhejiang Provincial CDC:** Min Yu, Ruying Hu, Hao Wang. Tongxiang CDC: Yijian Qian, Chunmei Wang, Kaixu Xie, Lingli Chen, Yidan Zhang, Dongxia Pan, Qijun Gu. **Hunan Provincial CDC:** Yuelong Huang, Biyun Chen, Li Yin, Huilin Liu, Zhongxi Fu, Qiaohua Xu. **Liuyang CDC:** Xin Xu, Hao Zhang, Huajun Long, Xianzhi Li, Libo Zhang, Zhe Qiu.

Contents for SI: 10 pages, 3 tables and 3 figures.

Table S1. Counts of incident cases for major cardiovascular diseases in 10 regions of CKB cohort.

Region	CVD	IHD	AMI	Stroke	Hemorrhagic stroke	Ischemic stroke	MACE	MVE	MCE
Qingdao	8,595	4,455	691	2,062	249	1,788	2,590	2645	692
Harbin	27,859	13,528	836	14,958	1,015	14,240	15,320	15,391	836
Haikou	8,278	2,023	427	4,514	234	4,353	4,735	4,738	427
Suzhou	6,149	1,391	134	2,952	448	2,437	2,978	3,061	135
Liuzhou	12,670	4,909	291	5,014	636	4,393	5,162	5,230	292
Pengzhou	12,619	3,929	352	3,614	848	2,825	3,830	3,889	352
Maiji	12,480	3,793	328	4,450	760	3,772	4,627	4,700	329
Huixian	19,531	6,425	769	9,964	1,422	8,770	10,470	10,567	781
Tongxiang	19,943	3,509	274	3,501	771	2,707	3,681	3,727	275
Liuyang	19,906	6,361	502	6,193	1,301	4,889	6,412	6,578	503
Total	148,030	50,323	4,604	57,222	7,684	50,174	59,805	60,526	4,622

Abbreviations: CVD, cardiovascular disease; IHD, ischemic heart disease; AMI, acute myocardial infraction; MACE, major adverse cardiovascular events; MVE, major vascular events; MCE, major coronary events.

Table S2. Annual counts (%) of cardiovascular incident cases in 10 regions of CKB cohort.

Region	Years of follow-up													Sum
	2005	2006	2007	2008	2009	2010	2011	2012	2013	2014	2015	2016	2017	
Qingdao	69	235	443	642	663	650	642	790	867	951	958	881	804	8595
	(0.80%)	(2.73%)	(5.15%)	(7.47%)	(7.71%)	(7.56%)	(7.47%)	(9.19%)	(10.09%)	(11.06%)	(11.15%)	(10.25%)	(9.35%)	(100.00%)
Harbin	189	526	1712	2782	2569	2616	2823	2537	2612	2500	2338	2398	2257	27859
	(0.68%)	(1.89%)	(6.15%)	(9.99%)	(9.22%)	(9.39%)	(10.13%)	(9.11%)	(9.38%)	(8.97%)	(8.39%)	(8.61%)	(8.10%)	(100.00%)
Haikou	54	176	234	455	553	750	866	987	1052	820	796	863	672	8278
	(0.65%)	(2.13%)	(2.83%)	(5.50%)	(6.68%)	(9.06%)	(10.46%)	(11.92%)	(12.71%)	(9.91%)	(9.62%)	(10.43%)	(8.12%)	(100.00%)
Suzhou	8	21	106	207	353	410	543	582	670	783	838	824	804	6149
	(0.13%)	(0.34%)	(1.72%)	(3.37%)	(5.74%)	(6.67%)	(8.83%)	(9.46%)	(10.90%)	(12.73%)	(13.63%)	(13.40%)	(13.08%)	(100.00%)
Liuzhou	69	349	650	979	1106	1142	1119	876	1429	1354	1235	1257	1105	12670
	(0.54%)	(2.75%)	(5.13%)	(7.73%)	(8.73%)	(9.01%)	(8.83%)	(6.91%)	(11.28%)	(10.69%)	(9.75%)	(9.92%)	(8.72%)	(100.00%)
Pengzhou	34	129	262	437	812	550	751	1501	1373	1972	1006	3065	727	12619
	(0.27%)	(1.02%)	(2.08%)	(3.46%)	(6.43%)	(4.36%)	(5.95%)	(11.89%)	(10.88%)	(15.63%)	(7.97%)	(24.29%)	(5.76%)	(100.00%)
Maiji	24	93	205	402	611	474	3856	2120	750	665	825	1239	1216	12480
	(0.19%)	(0.75%)	(1.64%)	(3.22%)	(4.90%)	(3.80%)	(30.90%)	(16.99%)	(6.01%)	(5.33%)	(6.61%)	(9.93%)	(9.74%)	(100.00%)

Huixian	121<br>(0.62%)	379<br>(1.94%)	581<br>(2.97%)	1116<br>(5.71%)	1452<br>(7.43%)	1585<br>(8.12%)	1485<br>(7.60%)	2365<br>(12.11%)	2362<br>(12.09%)	2362<br>(12.09%)	2191<br>(11.22%)	1847<br>(9.46%)	1685<br>(8.63%)	19531<br>(100.00%)
Tongxiang	32<br>(0.16%)	87<br>(0.44%)	6026<br>(30.22%)	5244<br>(26.29%)	4284<br>(21.48%)	308<br>(1.54%)	399<br>(2.00%)	472<br>(2.37%)	558<br>(2.80%)	589<br>(2.95%)	603<br>(3.02%)	624<br>(3.13%)	717<br>(3.60%)	19943<br>(100.00%)
Liuyang	4<br>(0.02%)	183<br>(0.92%)	392<br>(1.97%)	1761<br>(8.85%)	1773<br>(8.91%)	2080<br>(10.45%)	1782<br>(8.95%)	1819<br>(9.14%)	2086<br>(10.48%)	2043<br>(10.26%)	1841<br>(9.25%)	2423<br>(12.17%)	1719<br>(8.64%)	19906<br>(100.00%)
Total	604<br>(0.41%)	2178<br>(1.47%)	10611<br>(7.17%)	14025<br>(9.47%)	14176<br>(9.58%)	10565<br>(7.14%)	14266<br>(9.64%)	14049<br>(9.49%)	13759<br>(9.29%)	14039<br>(9.48%)	12631<br>(8.53%)	15421<br>(10.42%)	11706<br>(7.91%)	148030<br>(100%)

Table S3. Hazard ratios of cardiovascular diseases associated with per 10 μg/m^3 increase in $PM_{2.5}$ concentrations in different regions.

Regions	Unadjusted model	Model 1	Model 2	Model 3
Qingdao	1.22 (1.07, 1.39)	1.20 (1.05, 1.38)	1.23 (1.07, 1.40)	1.21 (1.05, 1.38)
Liuzhou	0.98 (0.95, 1.02)	0.98 (0.95, 1.02)	0.98 (0.95, 1.02)	1.00 (0.97, 1.05)
Haikou	0.95 (0.78, 1.15)	0.95 (0.78, 1.15)	0.94 (0.78, 1.14)	1.06 (0.79, 1.44)
Suzhou	1.05 (0.98, 1.12)	1.05 (0.98, 1.12)	1.05 (0.98, 1.13)	0.99 (0.92, 1.07)
Harbin	1.04 (1.01, 1.08)	1.04 (1.01, 1.08)	1.04 (1.01, 1.07)	1.04 (1.01, 1.08)
Pengzhou	0.98 (0.94, 1.02)	0.97 (0.93, 1.02)	0.98 (0.93, 1.03)	0.98 (0.94, 1.02)
Maiji	1.39 (1.20, 1.61)	1.40 (1.21, 1.63)	1.46 (1.26, 1.71)	1.39 (1.19, 1.64)
Liuyang	1.04 (0.94, 1.15)	1.04 (0.94, 1.15)	1.03 (0.93, 1.13)	0.99 (0.87, 1.12)
Tongxiang	1.64 (1.34, 2.01)	1.64 (1.34, 2.01)	1.69 (1.39, 2.06)	1.28 (1.06, 1.55)
Huixian	1.51 (1.30, 1.74)	1.50 (1.30, 1.73)	1.42 (1.24, 1.62)	1.34 (1.16, 1.54)

Notes: Unadjusted model, adjusting for age and sex only. Model 1, adjusting for age, sex, active smoking status, passive smoking status. Model 2, adjusting for age, sex, active/passive smoking status, education, BMI, self-rated health, alcohol consumption, physical activity, household income, solid fuel used for cooking/heating. Model 3, adjusting for age, sex, active/passive smoking status, education, BMI, self-rated health, alcohol consumption, physical activity, household income, solid fuel used for cooking/heating, ozone and temperature.

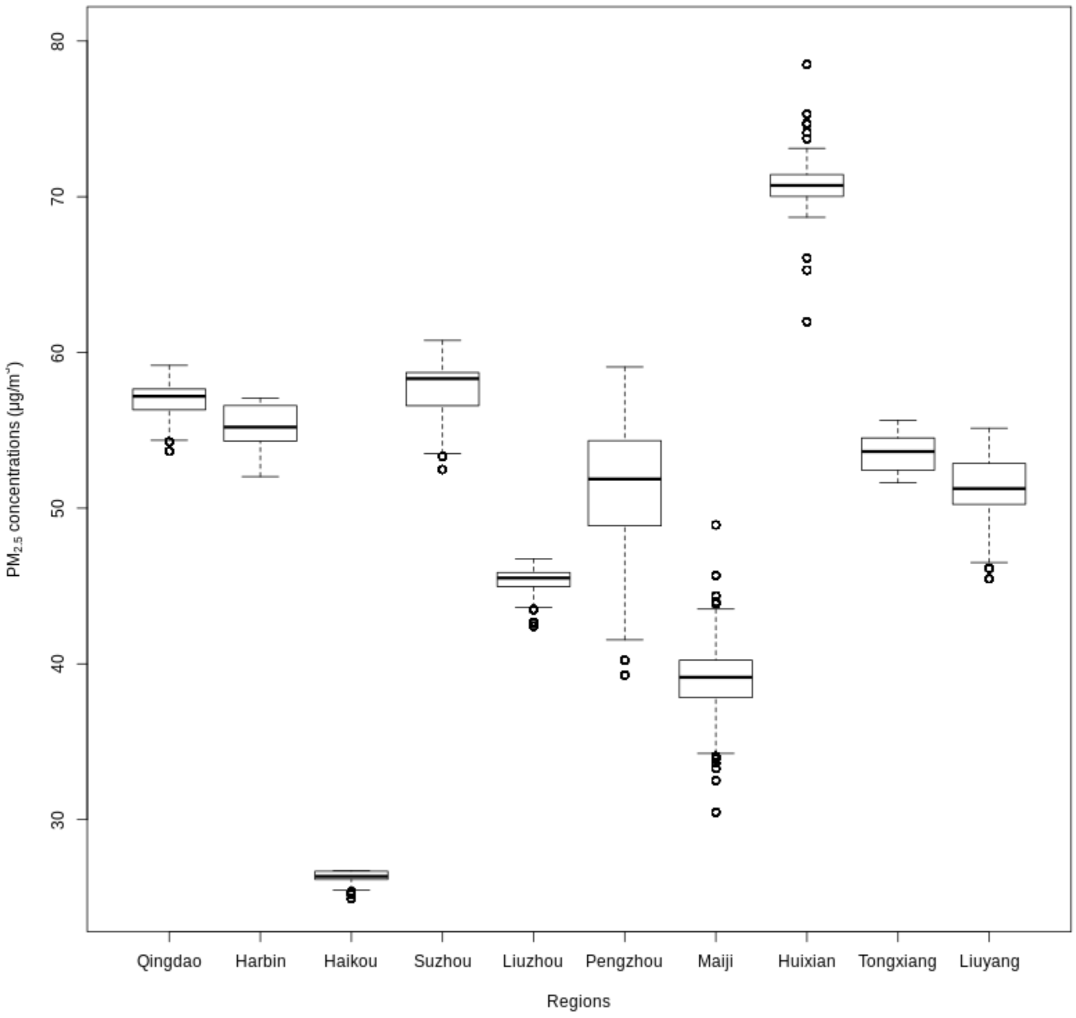

Figure S1. Boxplots for average annual mean concentrations of long-term exposure to $PM_{2.5}$ in 10 regions of CKB.

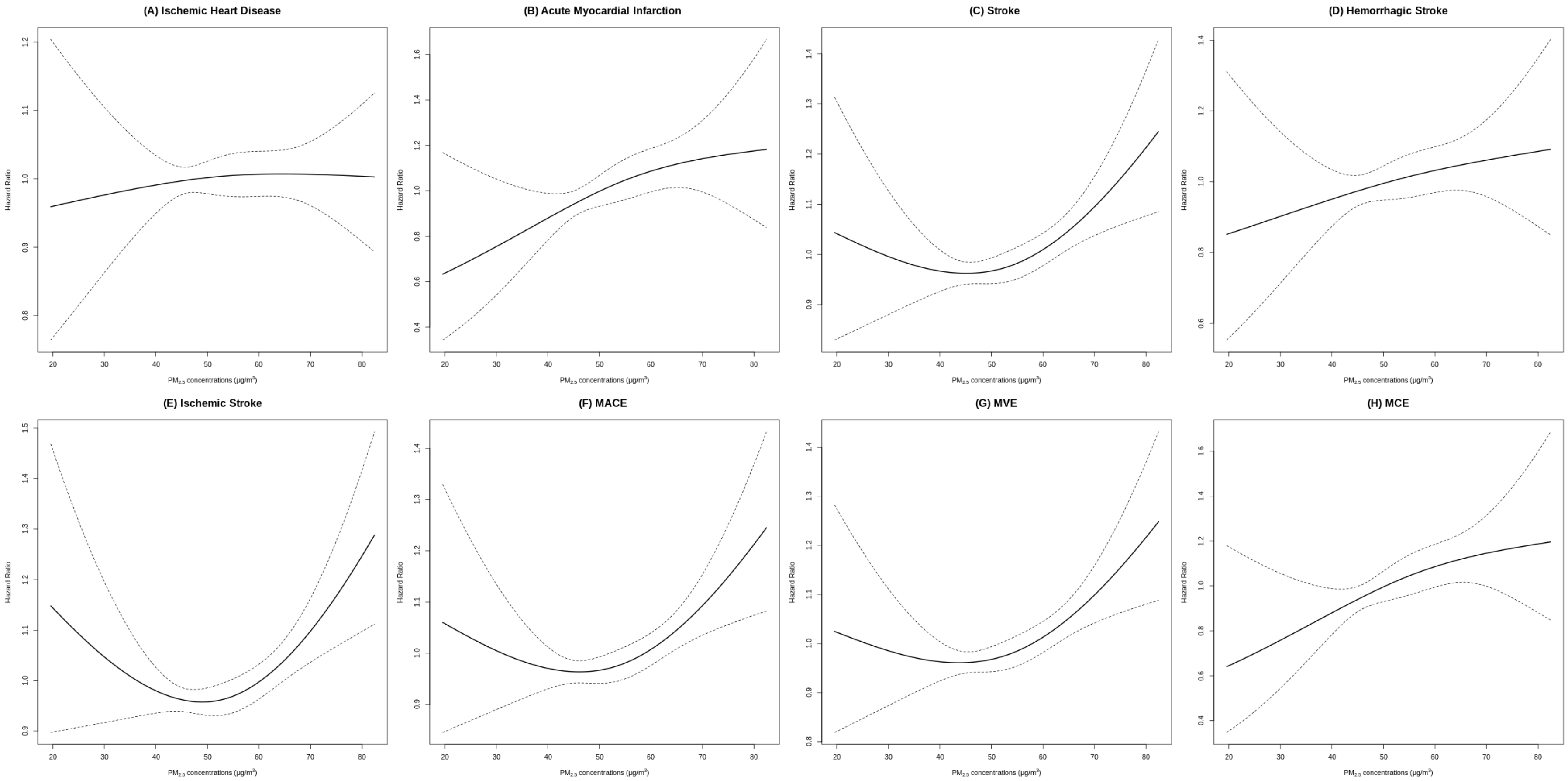

Figure S2. Concentration-response curves for long-term exposure to $PM_{2.5}$ and incident risk of specific cardiovascular diseases. (A) Ischemic heart disease, (B) Acute myocardial infarction, (C) Stroke, (D) Hemorrhagic stroke, (E) Ischemic stroke, (F) Major adverse cardiovascular events, (G) Major vascular events, (H) Major coronary events.

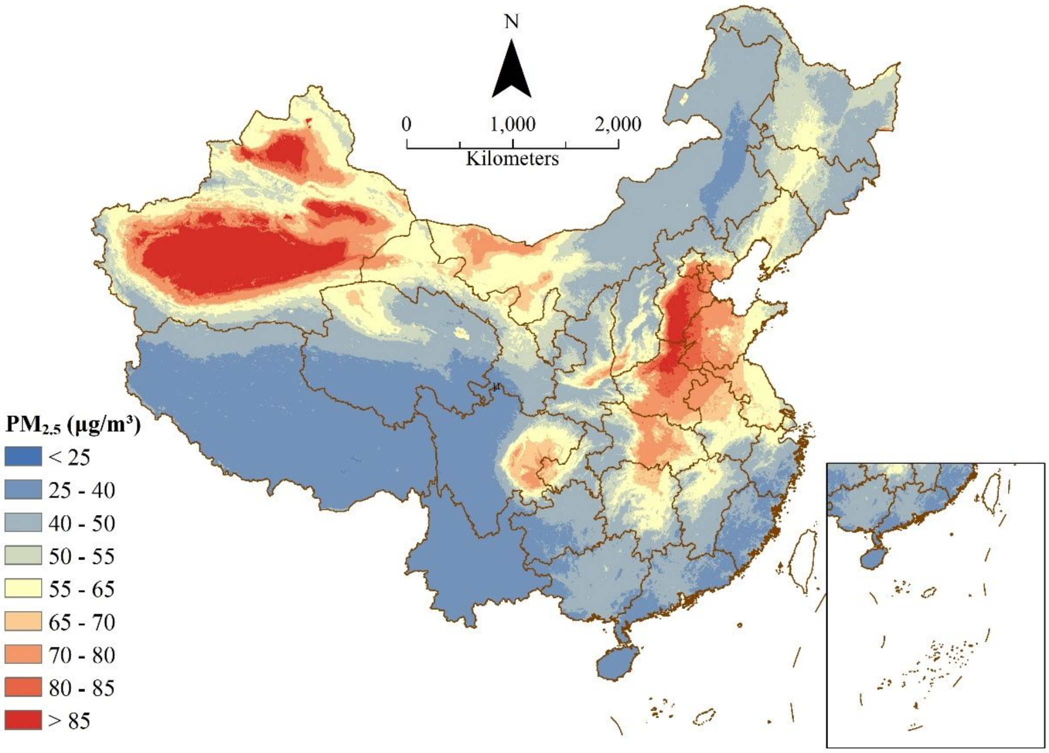

Figure S3. Predicted $PM_{2.5}$ concentration from the satellite-based exposure assessment model from 2005 to 2017.
